# Comparison of Disk Diffusion and Etest Methods to Determine the Susceptibility of *Staphylococcus aureus* Circulating in Riyadh, Saudi Arabia to Fusidic Acid

**DOI:** 10.1155/2012/391251

**Published:** 2012-07-24

**Authors:** Ali M. Somily, David R. Peaper, Elijah Paintsil, Thomas S. Murray

**Affiliations:** ^1^Department of Pathology, Microbiology Unit, College of Medicine, King Saud University and King Khalid University Hospital, Riyadh 11411, Saudi Arabia; ^2^Department of Laboratory Medicine, Yale School of Medicine, New Haven, CT 06510, USA; ^3^Pathology and Laboratory Medicine Service, Veteran Affairs (VA) Connecticut Healthcare System, West Haven, CT 06516, USA; ^4^Departments of Pediatrics and Pharmacology, Yale School of Medicine, New Haven, CT 06510, USA; ^5^Department of Medical Sciences, The Frank H. Netter MD School of Medicine, Quinnipiac University, Hamden, CT 06518-7908, USA

## Abstract

Fusidic acid is a common therapy for staphylococcal infections in Saudi Arabia, but reports have suggested high rates of resistance among clinical isolates. Susceptibility testing of *S. aureus* to fusidic acid is further complicated by the lack of consensus on mean inhibitory concentrations (MIC) and disk diffusion cutoffs to determine resistance. The purpose of this study was to determine the correlation between disk diffusion and Etest determined MIC susceptibility results in clinical isolates of *S. aureus* from a large academic hospital in Riyadh, Saudi Arabia. Our data demonstrate excellent correlation between Etest determined MIC and disk diffusion susceptibility data, using either previously proposed zone sizes of ≥21 mm as susceptible and ≤18 mm as resistant or the EUCAST recommended zone size of ≤24 mm for resistance, in an area with relatively high rates of fusidic acid resistance.

## 1. Introduction

 The story of fusidic acid can be likened to the proverbial saying “the stone the builders rejected has become the cornerstone.” Fusidic acid has been used in Europe as an antistaphylococcal agent since the 1960s [1]. With the increasing frequency of methicillin resistant *Staphylococcus aureus* (MRSA) worldwide, the need for more active anti-staphylococcal drugs is inevitable. Fusidic acid, with favorable pharmacokinetics and pharmacodynamics, has the potential to fill this niche. It is available in intravenous, oral, and topical preparations and is widely distributed through the body, including areas such as bone, joint fluid, prostate, and abscesses, when given parenterally [2]. Furthermore, it has excellent bioavailability and is active against both methicillin susceptible and resistant staphylococcus and does not show cross-resistance with other antibiotics [3]. 

Fusidic acid binds the bacterial ribosome, preventing polypeptide elongation and protein synthesis [4]. There have been reports of rapidly increasing fusidic acid resistance in *S. aureus* in the last decade from centers in countries where it is routinely used [5–7]. The mechanisms of resistance to fusidic acid have been ascribed to proteins encoded by a variety of genes (e.g., *fusA* and *fusB)* [8, 9]. These proteins mediate resistance by (i) alteration of elongation factor (chromosomally mediated), (ii) altering permeability (plasmid mediated), (iii) inactivation of enzymes, and (iv) efflux of fusidic acid [8, 10]. Interestingly, the epidemiology of fusidic acid resistance has been well studied and attributed to inappropriate usage as monotherapy and indiscriminate prescription practices [2, 11]. 

A confounding factor in determining resistance rates of fusidic acid is that there are differing standardized minimum inhibitory concentration (MIC) break points used to classify *S. aureus *as fusidic acid resistant. Some authors have proposed that isolates with MIC ≤ 1.0 *μ*g/mL are susceptible (*S*) and those with MIC ≥ 2.0 *μ*g/mL are resistant (*R*) while others have proposed an MIC ≤ 0.5 *μ*g/mL as the susceptible breakpoint [10, 12]. Most recently Jones et al. compared broth dilution, Etest MIC, and disk diffusion, and they proposed an MIC ≥ 4.0 *μ*g/mL as the interpretive break point for resistance and ≤1.0 *μ*g/mL for susceptibility [13]. For disk diffusion testing, EUCAST has set the 10 *μ*g fusidic acid zone size for resistance at 24 mm, while Skov et al. recently proposed ≤18 mm for resistance and ≥21 mm as susceptible interpretive break points [10, 12].

 Fusidic acid is a common therapy in Saudi Arabia for *S. aureus* infection, but in at least one location MRSA fusidic acid resistance rates approach 96% [14]. In this context, our study sought to determine the correlation of disk diffusion zone size and Etest MIC using different published criteria, in a setting of high *S. aureus* resistance in Riyadh, Saudi Arabia. These results will help inform the appropriateness of using only disk diffusion to determine *S. aureus* susceptibility to fusidic acid. 

## 2. Materials and Methods

### 2.1. Samples

One hundred and sixty *S. aureus* clinical specimens consecutively collected from January 1, 2009 to February 28, 2009 by the Clinical Microbiology Lab at King Khalid University Hospital, Riyadh, Saudi Arabia were studied. *S. aureus* was identified by colony morphology and the presence of *β*-hemolysis and confirmed by Gram stain, and positive catalase and StaphaurexPlus (Murex Biotech Ltd, Dartford, United Kingdom) reactions. 

Of the original 160 *S. aureus* isolates collected, 122 had complete data for both Etest MIC and disk diffusion susceptibility testing and were included in the study. These 122 isolates were recovered from 103 patients with either colonization (nasal swabs) or probable infection. Ninety-two patients provided single specimens that grew *S. aureus*, while 11 patients had multiple specimens (*n* = 2–7) with *S. aureus *accounting for the remaining 30 isolates. Since bacterial isolates from the same patient were recovered from independent clinical samples submitted to the microbiology laboratory and received separate susceptibility testing, they were included in the study. Isolates from presumed infections were recovered from soft tissue including pus, joint fluid, and blood samples. This study was approved by the Institutional Review Board of the College of Medicine, King Saud University. 

### 2.2. Susceptibility Testing

All susceptibility testing was carried out using Clinical and Laboratory Standards Institute recommendations [15]. MIC to fusidic acid were determined using Etests (BioMérieux, AB Biodisk, Solna, Sweden) on Mueller-Hinton agar incubated for 24 h. Disk diffusion zone sizes were determined by direct colony suspension to 0.5 McFarland, the suspension inoculated to Mueller-Hinton plates with 10 *μ*g fusidic acid disks (Biomerieux, AB Biodisk, Solna, Sweden), and the plate was read after incubation for 16–20 h at 35°C. MRSA isolates were detected using either cefoxitin disk diffusion or oxacillin Etest assays. A colony suspension equivalent to 0.5 McFarland was inoculated to Mueller-Hinton agar with a 30 *μ*g cefoxitin disk(Oxoid, Basingstoke,UK) and interpreted after 16–20 h. MRSA was identified using a breakpoint of ≤21 mm zone size for cefoxitin disks. For the oxacillin Etests (AB Biodisk, Solna, Sweden), a 0.5 McFarland direct colony suspension was inoculated to Mueller-Hinton plates with 2.0% NaCl and interpreted after 24 hr incubation. An isolate with an MIC ≥ 4.0 *μ*g/mL was considered oxacillin resistant [15]. 

### 2.3. Statistical Analysis

Categorical data were compared using Fisher's exact test using Prism (GraphPad Software, La Jolla, CA). A *P* value of <0.05 was considered significant. 

## 3. Results

The patient population was 42% (50/119) female and 58% (69/119) male with no gender listed for three patients. There were 26.2% (32/122) isolates from nasal swabs, and 73.8% (90/122) isolated recovered from presumed sites of infection. MRSA represented 37.7% (46/122) of the total isolates. The rate of fusidic acid resistance among all *S. aureus* isolates was 36.9% (45/122) using an MIC break point of ≥4.0 *μ*g/mL and 40.2% (49/122) using a break point of ≥2.0 *μ*g/mL for resistance determination (Table 1). Using a breakpoint ≥2.0 *μ*g/mL for resistance determination, fusidic acid resistance was significantly higher amongst MRSA isolates at 80.4% (37/46) compared with MSSA 15.8% (12/76) (Table 1, *P* < 0.001). This high rate of resistance is consistent with previously published data from this institution on fusidic acid resistance amongst MRSA strains [14]. Using the same breakpoint of 2.0 *μ*g/mL, rates of fusidic acid resistance were lower for isolates recovered from nasal swabs, 18.8%, (6/32) compared with isolates recovered from other sites, 47.7% (43/90) (*P* < 0.0058). 

**Figure 1:**
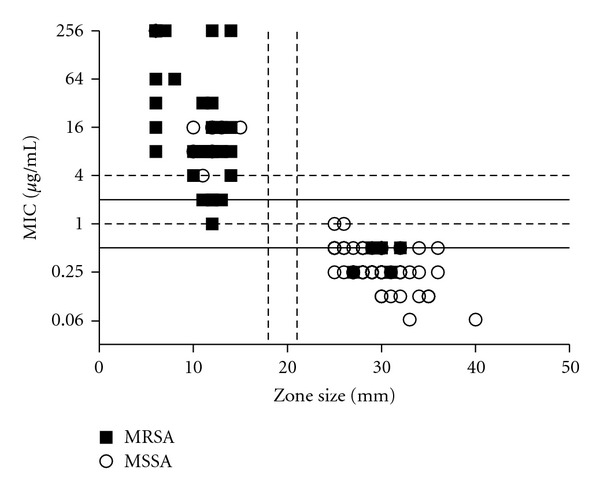
Semi log scatterplot of fusidic acid MIC and zone size. MIC is shown on a log(2) scale. Horizontal dashed lines (- - -) correspond to the MIC interpretive criteria of Jones et al., and solid lines (-) correspond to MIC interpretive criteria of Skov et al. Vertical dashed lines (- - -) correspond to zone diameters of 18 mm and 21 mm proposed by Skov et al. MRSA (■) and MSSA (○) isolates are shown.

Regression analysis of a semilog scatterplot revealed a strong correlation (83.8%) between Etest determined MIC and disk diffusion susceptibility methods (Figure 1). Disk diffusion criteria using zone sizes of ≥21 mm as susceptible and ≤18 mm as resistant produced no isolates in the intermediate range for disk diffusion interpretation [15]. In fact, the smallest zone size to fall in the susceptible range was 25 mm, and the largest zone size in the resistant category was 15 mm, offering a clear dichotomy between susceptible and resistant organisms using the disk diffusion criteria proposed by Skov et al. These disk diffusion results also correlate when the EUCAST zone size cutoff of 24 mm is applied, with no discrepant isolates comparing the two disk diffusion criteria [12]. Thus, applying either of these disk diffusion criteria to the data gives a fusidic acid resistance rate of 41.0% (50/122) (Table 2). 

When the Skov et al. MIC criteria were applied (MIC of ≤0.5 *μ*g/mL = *S* and ≥2.0 *μ*g/mL = *R*) the correlation between either disk diffusion cut-offs and Etest MIC was 100% for susceptible (70/70) and resistant (50/50) MIC criteria [10]. There were three strains that were intermediate applying the Skov et al. MIC criteria (Table 2). Two of these isolates with MIC = 1.0 *μ*g/mL were susceptible by disk diffusion, while one isolate with an MIC = 1.0 *μ*g/mL was resistant by disk diffusion using either the Skov et al. or EUCAST criteria (Table 2). When the Jones et al. criteria were applied (MIC ≤ 1.0 *μ*g/mL = *S* and MIC ≥ 4.0 *μ*g/mL = R) there was 99% (72/73) correlation with disk diffusion for susceptible strains [13]. One isolate with an MIC = 1.0 *μ*g/mL was classified as resistant by disk diffusion testing (12 mm) but was susceptible when applying the Jones et al. criteria. For resistant strains there was 100% correlation (46/46) between MIC and disk diffusion. There were four strains in the intermediate range (MIC = 2.0 *μ*g/mL) that were resistant by disk diffusion testing (zones sizes from 11 mm–13 mm) (Table 2). For these isolates disk diffusion results best correlated with the application of the Skov et al. criteria for MIC interpretation. 

## 4. Discussion 

Careful studies of broth dilution, Etest MIC determination, and disk diffusion have demonstrated excellent correlation in measuring *S. aureus* resistance to fusidic acid, but these studies have led to slightly different interpretive criteria for classifying resistance [10, 12, 13]. Since broth dilution is not frequently performed in most clinical microbiology laboratories because of its laborious nature, we sought to determine the correlation between Etest MIC and disk diffusion for measurement of *S. aureus* resistance to fusidic acid in isolates from an academic hospital in Riyadh, Saudi Arabia. We analyzed *S. aureus* recovered from both nasal swabs and potential sites of infection, and our study set contained both MSRA and MSSA isolates. Consistent with previous studies, our data further confirm the strong correlation between disk diffusion and MIC regardless of the interpretative criteria used [10, 12, 13, 16]. 

A high percentage of *S. aureus* isolates in this study were resistant to fusidic acid (36.7%–41%) regardless of which MIC or disk diffusion breakpoint criteria was applied, suggesting our data is relevant to areas where fusidic acid resistance is frequently encountered [14]. However, the isolates were collected over a short-time period and in some cases from the same patient, so it is likely some isolates were clonal. Therefore, the applicability of these results to other institutions depends in part on the local, circulating *S. aureus* strains. Only isolates with MIC of either 1.0 *μ*g/mL or 2.0 *μ*g/mL (7/123, 6%) gave discrepant results compared with disk diffusion testing. One isolate with an MIC of 1.0 *μ*g/mL considered susceptible by the Jones et al. criteria and intermediate by the Skov et al. criteria was resistant (zone size 12 mm) by disk diffusion testing. This is the only isolate that also gives a discrepant result between disk diffusion and MIC if the MIC EUCAST criteria are applied wherein an MIC of ≤1.0 *μ*g/mL is considered susceptible. 

We did not perform broth dilution in this study because it is not a standard procedure in most clinical microbiology laboratories while Etest and disk diffusion testing are widespread. Importantly, we identified that disk diffusion testing results correlated well regardless of the MIC interpretive criteria applied: EUCAST (one discrepancy), the criteria of Skov et al. (three discrepancies) and that of Jones et al. (five discrepancies). This suggests disk diffusion, regardless of whether the EUCAST or breakpoints proposed by Skov et al. are applied, is both a cost effective and reliable way to perform initial susceptibility testing of *S. aureus* to fusidic acid in areas where resistant organisms are frequently encountered.

## Figures and Tables

**Table 1:** Comparison of MIC interpretive criteria for *S. aureus* and fusidic acid.

MIC *μ*g/mL	≤0.5 (*n* = 70)	1.0 (*n* = 3)	2.0 (*n* = 4)	≥4.0 (*n* = 45)	Totals
Breakpoints proposed by Jones et al. [13]	Susceptible		Intermediate	Resistant	
Breakpoints proposed by Skov et al. [10]	Susceptible	Intermediate	Resistant		
MSSA isolates	62	2	0	12	76
MRSA isolates	8	1	4	33	46

Totals	70	3	4	45	122

**Table 2:** Comparison of disk diffusion and proposed MIC interpretive criteria for *S. aureus *and fusidic acid.

MIC *μ*g/mL	≤0.5 (*n* = 70)	1.0 (*n* = 3)		2.0 (*n* = 4)	≥4.0 (*n* = 45)
Breakpoints proposed by Jones et al. [13]	Susceptible			Intermediate	Resistant
Breakpoints proposed by Skov et al. [10]	Susceptible	Intermediate		Resistant	
Disk diffusion result^∗^	Susceptible		Resistant		
zone size (mm)	≥25	25, 26	12	11, 12, 12, 13	≤15

^∗^The results from disk diffusion are the same using either the EUCAST criteria (≤24 mm = *R*) or zone size breakpoints by Skov et al. (≤18 mm = *R*, ≥21 mm = *S*).
